# Vitamin D and Endometriosis: Is There a Mechanistic Link?

**DOI:** 10.1002/cbf.70037

**Published:** 2024-12-31

**Authors:** Bethany Scout Jennings, Martin Hewison

**Affiliations:** ^1^ Department of Metabolism and Systems Science, School of Medical Sciences University of Birmingham Birmingham UK; ^2^ Centre for Endocrinology, Diabetes and Metabolism Birmingham Health Partners Birmingham UK

**Keywords:** endometriosis, immune, inflammation, steroidogenic, vitamin D

## Abstract

Endometriosis is a prevalent chronic gynaecological disorder, but its cause is still unclear, and both genetic and environmental factors may contribute disease aetiology. Prominent amongst the latter is vitamin D which can be obtained either by the action of sunlight on skin or from dietary sources. Serum levels of the main circulating form of vitamin D, 25‐hydroxvitamin D (25(OH)D), have been reported to be inversely correlated with endometriosis, suggesting that vitamin D‐deficiency may be a risk factor for the disease. Crucially, the active form of vitamin D, 1,25‐dihydroxyvitamin D (1,25(OH)_2_D) is known to exert many functions beyond its established role in the endocrinology of mineral homoeostasis and prevention of rickets. Several of these extra‐skeletal effects of 1,25(OH)_2_D may impact the risk and progression of endometriosis. The following review details the studies that have assessed associations between vitamin D status/supplementation and endometriosis severity and disease progression, but also describes the mechanistic targets for 1,25(OH)_2_D in endometriosis with specific reference to immunomodulatory responses and effects on angiogenesis. Endometriosis is an under‐reported health issue with poor non‐invasive options for diagnosis. Given that vitamin D‐deficiency may trigger or exacerbate key pathophysiological responses linked to endometriosis, analysis of vitamin D status in women may provide an alternative risk marker for endometriosis. Treatment options for endometriosis are also limited and the review will also consider whether vitamin D supplementation has a role in the management of endometriosis, either in prevention or treatment.

## Summary

- Vitamin D has been linked to a wide range of human health conditions beyond its established role in calcium and bone homoeostasis.
- One of the less well documented facets of extra‐skeletal vitamin D is the possible link between vitamin D‐deficiency and the reproductive disorder endometriosis.
- The current review aims to address this by detailing the studies that have linked vitamin D to endometriosis, as well as describing the plausible mechanistic basis for this link.
- This review is important because endometriosis is a prevalent disorder but monitoring and treatment options for the disorder are poor.
- Vitamin D may provide an alternative strategy for the management of endometriosis.

## 1 Introduction

Endometriosis is a chronic gynaecological disease defined by the presence of endometrial tissue outside the uterine cavity, mostly in the pelvis [1]. As oestrogen and progesterone morphologically change endometrial tissue, the signs and symptoms of endometriosis are influenced by these sex hormones during the menstrual cycle [2]. There is huge variability in the presentation of endometriosis. Common symptoms include dysmenorrhoea, which disrupts daily activities, chronic pelvic pain, cyclical haematuria, cyclical dyschezia, sexual intercourse‐associated pain and infertility [3, 4]. It is estimated that approximately 10% of UK women of reproductive age have endometriosis [5], making it the UK's second most common gynaecological disease.

Although endometriosis is a prevalent disorder, it is under‐reported, under‐diagnosed, costly to manage, and poorly understood. Many women do not recognise their chronic pelvic pain as pathological, especially as it is pain related to the menstrual cycle. Menstrual‐related problems are often trivialised or taboo, preventing women from seeking professional help [6]. Endometriosis‐related pain may be dismissed as normal dysmenorrhea, and the high variability of clinical presentation often causes misdiagnosis and delayed referral, leading to an average diagnostic delay of 6.7 years [7]. One study found that half of the general practitioners interviewed were unable to list 3 of the main symptoms of endometriosis, highlighting poor understanding of the condition [8]. Although, pathophysiologically, endometriosis is defined by the presence of endometrial tissue at ectopic sites, the exact aetiology of the disorder is still unknown, and is believed to be a multifactorial condition associated with both genetic [9] and environmental [10] factors. Key abnormalities associated with endometriosis include retrograde menstruation, metaplasia, lymphatic and circulatory dissemination and immune dysfunction [1].

The most popular theory for the origin of endometriosis is that retrograde menstruation, the flow of menstrual blood into the pelvic cavity through the fallopian tube, facilitates the implantation and survival of endometrial tissue in extrauterine pelvic tissue [11]. This is endorsed by the high correlation between endometriosis and a prolonged menses, and the close proximity of the most common ectopic endometrial sites—the ovaries and uterosacral ligaments—to the openings of the fallopian tubes. In support of this pathophysiological origin for endometriosis, one reported that 97% of women with endometriosis had retrograde spillage [12]. However, it is important to recognise that the establishment of ectopic endometriotic lesions is likely facilitated through a combination of retrograde menstruation and immunological or biochemical dysfunction, for example, a defect in immune surveillance or detoxification processes [13]. The aim of this review is to discuss how one particular environmental factor—vitamin D—could influence the pivotal pathophysiological mechanisms associated with endometriosis, and the potential implications of these links for the management of this debilitating disease.

## 2 Vitamin D

Vitamin D is an umbrella term for a group of essential, multifunctional secosteroids. Around 90% of vitamin D is synthesised endogenously in the epidermis of the skin following exposure to ultraviolet B (UVB) light, with the remainder being obtained from the diet, notably from vitamin D‐rich foodstuffs such as oily fish [14]. There are two main forms of vitamin D: vitamin D3 (D3) and vitamin D2 (D2). The latter is obtained from plant‐based dietary intake, whereas D3 is derived primarily from skin exposure to UVB [15]. Both D2 and D3 are used as vitamin D supplements [16]. Both dietary (including supplementary) and UVB‐generated vitamin D undergo initial metabolism in the liver to form 25‐hydroxyvitamin D (25(OH)D), the major circulating form of vitamin D [14]. Serum 25(OH)D is the major circulating marker of vitamin D status and is routinely used to define vitamin D‐deficiency or ‐sufficiency. However, the precise concentrations of 25(OH)D that indicate vitamin D‐sufficiency are still subject to debate. In the UK, the Science Advisory Council on Nutrition (SACN) have proposed that values below 25 nmol/L (10 ng/mL) represent vitamin D‐deficiency [17]. By contrast, in North America, both the Institute of Medicine (now National Academy of Medicine) [18], and more recently the Endocrine Society [19, 20] have proposed 50 nmol/L (20 ng/mL) as the target for vitamin D‐sufficiency.

After synthesis in the liver, 25(OH)D is transported throughout the body bound to vitamin D binding protein (DBP), a multifunctional glycoprotein produced in the liver [21]. DBP plays a key role in synthesis of the active form of vitamin D, 1,25‐dihydroxyvitamin D (1,25(OH)_2_D) via megalin‐mediated endocytic uptake of DBP and its 25(OH)D cargo from glomerular filtrate into proximal tubule cells within the kidney [22]. This renal handling of DBP and 25(OH)D leads to the conversion of 25(OH)D to 1,25(OH)_2_D mediated by the enzyme 25‐hydroxyvitamin‐1α‐hydroxylase (1α‐hydroxylase) [22]. Endocytic uptake of 25(OH)D also occurs in other megalin‐expressing tissues. However, for most target tissues, the acquisition of 25(OH)D and 1,25(OH)_2_D appears to occur via passive diffusion of the fraction of these metabolites that are not bound to DBP, consistent with the free hormone hypothesis [22]. Following entry into target cells, 25(OH)D may be converted to active 1,25(OH)_2_D if these cells exhibit 1α‐hydroxylase activity [23]. In the absence of extra‐renal 1α‐hydroxylase, cellular responses to vitamin D are dependent on uptake of active 1,25(OH)_2_D itself, with functional responses being driven following binding of 1,25(OH)_2_D to the nuclear vitamin D receptor (VDR) [24]. By modulating transcriptional and epigenetic responses at thousands of loci throughout the genome, the 1,25(OH)_2_ D‐VDR complex is able to regulate a wide range of biological activities [24]. Conventionally, much of this relates to the calciotropic effects of 1,25(OH)_2_D to maintain mineral homoeostasis and skeletal health, but in recent years there has been growing interest in the extra‐skeletal effects of vitamin D [25]. The current review will detail how some of these non‐classical actions of vitamin D have the potential to influence one particular extra‐skeletal disorder, namely endometriosis.

## 3 Vitamin D and Endometriosis

### 3.1 Serum Vitamin D Status and Endometriosis

The ability of vitamin D to influence biological functions beyond its classical target tissues such as the gastrointestinal tract (dietary mineral uptake) and the skeleton (bone turnover) stems primarily from the ubiquitous expression of VDR in tissues throughout the body [26]. Coupled to this, a wide range of reports have described diverse biological responses to 1,25(OH)_2_D in these VDR‐expressing tissues, suggesting that the vitamin D system is involved in healthy immune function, angiogenesis, gastrointestinal homoeostasis, muscle function, glucose homoeostasis, cell metabolism and many other pivotal physiological actions [25]. However, the broader functional impact of vitamin D beyond the skeleton is also supported by the links between vitamin D and many human health issues other than its classical role in protecting against bone disease rickets [27]. Most prominently, serum vitamin D (25(OH)D) deficiency has been shown to be associated with both risk and severity of many diseases [14], the implication being that impaired 25(OH)D leads to decreased availability and VDR‐mediated biological responses to 1,25(OH)_2_D. Other evidence of the role of vitamin D in disease or health issues can arise from in vitro/ex vivo experiments, animal modelling or vitamin D supplementation studies.

Table 1 shows the range of different types of evidence that has been reported for a link between vitamin D and endometriosis. Of the 20 reported studies, 11 assessed the relationship between serum 25(OH)D or serum 1,25(OH)_2_D and endometriosis symptoms, and six assessed the impact of vitamin D supplementation on endometriosis. A further six studies assessed levels of expression or single‐nucleotide polymorphism (SNP) variations for DBP or VDR, and how these variations impact endometriosis. Of the 12 association studies that assessed the link between vitamin D status and endometriosis, eight reported increased disease risk and disease severity in women with lower levels of 25(OH)D [28, 29, 30, 31, 32, 33, 34, 35]. Of the remaining studies, two showed no effect of serum 25(OH)D levels [36, 37], whilst one showed apparent high levels of 25(OH)D in women with endometriosis [38]. In one of the studies where there was no association between serum 25(OH)D and endometriosis, serum levels of the active form of vitamin D, 1,25(OH)_2_D, were shown to be higher [37]. In general, studies of vitamin D and endometriosis have restricted measurements of vitamin D status to 25(OH)D alone, but in this case several metabolites, including 1,25(OH)_2_D, were also analysed. The elevated levels of 1,25(OH)_2_D in women with endometriosis may reflect increased extra‐renal 1α‐hydroxylase within endometriosis ovarian tissue as described for other inflammatory conditions [48]. Reported studies have shown increased expression of the gene for 1α‐hydroxylase (*CYP27B1*) in ovarian tissue from women with endometriosis [49], although the extent to which any local synthesis of 1,25(OH)_2_D in this tissue could spill over into the general circulation is unclear.

**Table 1:** Publications describing links between endometriosis and vitamin D.

References	# patients/controls	Serum 25(OH)D and 1,25(OH)_2_D levels
[28]	70,556	Nurses' Health Study II. Higher predicted 25(OH)D associated with lower risk of endometriosis.
[29]	54/56	Serum (12 ng/mL) and PF (4 ng/mL) 25(OH)D lower in women with endometriosis vs. no endometriosis controls (22 ng/mL and 6 ng/mL respectively).
[30]	135/90	Low serum 25(OH)D more frequent in women with endometriosis (80% vs. 33.3%) and associated with moderate to severe pelvic pain.
[31]	49	42 women (85.7%) were 25(OH)D deficient (< 75 nmol/L). Inverse correlation between 25(OH)D and diameter of endometriomas.
[32]	440/30	Lower 25(OH)D levels associated with greater risk of genital endometriosis (GE).
[33]	16/16	Lower total 25(OH)D (9.55 ng/mL) with endometriosis vs. healthy (16.48 ng/mL). No difference in serum bioavailable or free 25(OH)D levels.
[34]	39/37	Lower serum 25(OH)D in severe endometriosis (17.2 ng/mL) compared to mild endometriosis (21.5 ng/mL) or healthy controls (21.8 ng/mL).
[34]	39/37	1,25(OH)_2_D levels not different between women with severe endometriosis and mild endometriosis or healthy controls.
[35]	16/16	Total, free and bioavailable 25(OH)D were lower in women with advanced endometriosis (*n* = 7) vs. healthy controls (*n* = 16).
[36]	217/217	No association between serum 25(OH)D in women with (17.9 ng/mL) and without (18.4 ng/mL) endometriosis.
[37]	42/113	Women with endometriosis had higher serum 1,25(OH)_2_D compared with healthy controls, but no difference for 25(OH)D.
[38]	87/53	Serum 25(OH)D higher in endometriosis (24.9 ng/mL) vs. healthy women (20.4 ng/mL) (*p* = 0.05) and positive association with disease severity.

	# patients treated/placebo	Vitamin D supplementation
[32]	240/200	Vitamin D (variable dose, 1000–10,000 IU/day, depending on baseline level) amplified reduction in pain and psycho‐emotional stabilisation in patients with genital endometriosis compared to standard therapy.
[39]	30/30	Vitamin D supplementation (50,000 IU/2 week, 12 weeks) reduced pelvic pain in patients with endometriosis compared to placebo.
[40]	17/17	Vitamin D supplementation (50,000 IU/week, 12–14 weeks) reduced β‐catenin protein in endometrial cells.
[41]	17/17	Vitamin D supplementation (50,000 IU/week, 12–14 weeks) reduced CD44 expression (CD44 is upregulated in endometriosis).
[42]	19/20	Vitamin D supplementation (50,000 IU/week, 12 weeks) did not reduce the severity of pelvic pain or dysmenorrhea after ablative surgery.
[43]	27/22	Vitamin D supplementation (2000 IU/day, 6 months) reduced pelvic pain in the endometriosis group but placebo showed similar changes.

	# patients/controls	Vitamin D binding protein (DBP) and vitamin D receptor (VDR) expression
[33]	16/16	No significant differences in DBP levels between endometriosis and control groups (serum 25(OH)D was lower in endometriosis).
[35]	16/16	Serum DBP levels were not associated with the severity of endometriosis.
[44]	56/20	Higher serum levels of the GC2 allele form of DBP in women with endometriosis (no control group women expressed only GC2 DBP).
[45]	57/38	Higher urinary DBP levels (2.16‐fold) associated with endometriosis.
[46]	13/6	Higher DBP (approximately fourfold) levels found in ectopic endometrial tissue compared to normal endometrial tissue.
[47]	132/133	No association between VDR polymorphisms in women with endometriosis vs. healthy controls or women with idiopathic infertility (*n* = 62).

The over‐arching conclusion from these observational studies is that endometriosis is frequently associated with lower serum vitamin D status relative to control subjects, and subsequent sections of this review will explore the potential impact of low serum concentrations of 25(OH)D on mechanisms associated with endometriosis disease pathogenesis. However, not all of these studies explicitly assessed the impact of vitamin D deficiency on endometriosis. When defined as serum 25(OH)D levels less than 50 nmol/L, a study of Iranian women reported an increased risk of endometriosis in vitamin D deficient women (Odds Ratio 2.7) compared to vitamin D sufficient women (serum 25(OH)D > 50 nmol/L) [29]. Other studies assessed the impact of vitamin D sufficiency/deficiency on disease severity. In a study of 104 women with endometriosis, vitamin D deficiency (serum 25(OH)D < 50 nmol/L) was more common in women with pelvic pain relative to those without pelvic pain (80% vs. 33%) [30]. Thus, whilst there is still disagreement concerning the precise target serum 25(OH)D level for vitamin D sufficiency in the general population, it would appear that serum levels > 50 nmol/L 25(OH)D are advantageous in the setting of endometriosis.

It is important to recognise that associations between serum 25(OH)D levels and endometriosis are not conclusive evidence of a causal role for vitamin D in this disease. A more useful method of determining whether vitamin D is involved in the pathogenesis of endometriosis would be to measure vitamin D levels before disease onset. One particular prospective cohort study used a predicted plasma 25(OH)D score based on a linear regression model which was previously created by correlating the plasma 25(OH)D concentrations of 1498 patients with factors such as their age, race, dietary vitamin D intake and alcohol intake. The study followed 70,556 premenopausal women without a diagnosis of endometriosis, and found that over a period of 14 years, 1385 of the women had an incident laparoscopically‐confirmed diagnosis of endometriosis [28]. The study found that participants with the top 20% of predicted serum 25(OH)D scores had 24% less of a risk of endometriosis than the women in the lowest 20% of predicted serum 25(OH)D levels. The predicted vitamin D score is useful because unlike obtaining a measurement from a single blood sample, it can represent a long‐term average which could be determined before diagnosis. Additionally, this study incorporated a large sample size, which facilitated clear statistical significance of data (*p* = 0.004) [28]. A limitation of using predicted vitamin D scores is that some predictions may not be completely accurate. However other studies using this model have validated its accuracy [50, 51], and it is also important to recognise that most other association studies rely on a single measurement of serum 25(OH)D to define disease risk or severity that may take years to develop. Another potential limitation of the predicted vitamin D study is that endometriosis diagnoses that were incidentally found due to infertility rather than abdominal pain were not included. This could have skewed results as different presentations of endometriosis could be affected differently by vitamin D.

The gold standard for functional links between vitamin D status and human disease is the assessment of disease impact following enhanced vitamin D status due to vitamin D supplementation. There have been relatively few randomised control trials (RCT) for vitamin D and endometriosis and reported studies have focused entirely on the impact of vitamin D supplementation and endometriosis symptoms. In one RCT, 60 women with endometriosis were randomised to receive either 50,000 IU vitamin D or placebo every 2 weeks for 12 weeks. Supplementation with vitamin D had the effect of raising serum 25(OH)D levels from 60 nmol/L at baseline to 90 nmol/L after 12 weeks, with no change observed in the placebo arm. The elevated serum 25(OH)D in the supplemented women decreased clinical markers, notably pelvic pain, consistent with a positive effect of vitamin D on endometriosis severity [39]. Other supplementation studies have shown positive effects of increased vitamin D on specific disease markers for endometriosis such as β‐catenin activity [40] and the cluster designation (CD) endometrial marker CD44 [41]. In another RCT, vitamin D supplementation reduced pelvic pain in women with endometriosis, but a similar reduction was also observed in the placebo arm of this study [43]. In this case, it was notable that the mean serum 25(OH)D levels at baseline were approximately 80 nmol/L, strongly suggesting that the women in this study were already vitamin D sufficient before the RCT [43]. In another study where vitamin D supplementation was reported to have no effect on pelvic pain [42], it is important to recognise that in this study vitamin D supplementation was carried out in women who were also receiving ablation surgery, and there were no recorded serum vitamin D levels at baseline or following supplementation [42].

### 3.2 Vitamin D Transport, Metabolism and Signalling in Endometriosis

In common with other human diseases, a link between vitamin D and endometriosis has also been explored through analysis of the expression of and genetic variation within components of the vitamin D system. The relatively high circulating levels of the serum vitamin D binding protein (DBP) means that this protein has been measured in a variety of endometriosis settings. Initial studies reported lower levels of DBP in peritoneal fluid (but not plasma) of women with endometriosis compared to controls [52]. By contrast, urinary DBP levels have been reported to be higher in women with endometriosis, relative to healthy controls [45]. Other studies have investigated possible correlations between the severity of endometriosis and serum DBP levels [35]. Whilst this study showed lower serum 25(OH)D levels in women with severe endometriosis compared to healthy controls, there was no significant difference in DBP levels [35]. The possible use of DBP as a marker of the progression of endometriosis has also been assessed, but although serum and peritoneal fluid DBP levels showed wide variations between women, there was no significant difference between women with suspected or confirmed endometriosis [53]. In an unbiased analysis of proteins from normal and endometriosis endometrial tissue using two‐dimensional electrophoresis, DBP was identified as one of 16 proteins with increased expression in disease tissue [46]. The overarching conclusion from these studies is that levels of DBP show changes in endometriosis, but the nature of these changes is different at endometrial tissue level versus peritoneal fluid or plasma. As such, further research is required to determine its value as a marker for endometriosis.

Measurement of serum levels of DBP is relatively straightforward using commercially available ELISA kits. By contrast, the tissue‐localisation of VDR means that the assessment of the expression of the pivotal intracellular receptor for 1,25(OH)_2_D is far more complex. One study that assessed mRNA expression for various vitamin D‐related factors including *VDR*, *CYP27B1* and the catabolic enzyme 24‐hydroxylase (*CYP24A1*) in ovarian tissues showed that women with endometriosis had higher expression of *VDR*, *CYP27B1* and *CYP24A1* [49]. It is unclear what this means for vitamin D function in endometriosis ovarian tissue, as higher *CYP27B1* and *VDR* would be consistent with improved 25(OH)D conversion to 1,25(OH)_2_D and subsequent signalling. By contrast this may then be negated by enhanced expression of the negative feedback catabolic enzyme *CYP24A1*. Further studies are required to determine actual changes in VDR protein expression and local activities of the two vitamin D enzymes.

### 3.3 Genetic Variations Within the Vitamin D System

Although analysis of DBP, VDR, 1α‐hydroxylase and 24‐hydroxylase provides a ‘snapshot’ of the vitamin D system in different endometriosis settings, it is not easy to obtain the tissue required to make these types of measurement. Other approaches to assessing the impact of the vitamin D system in different diseases is through analysis of well‐established genetic variations within the vitamin D system. Single nucleotide polymorphisms (SNPs) in genes associated with vitamin D transport, metabolism and signalling have been linked to inherited variations in different facets of vitamin D function. This includes an important contribution to the genetically‐determined component of circulating levels of 25(OH)D [54, 55] which have, in turn, enabled Mendelian Randomisation (MR) analyses to explore the possible impact of lifetime exposure to higher or lower levels of 25(OH)D and human disease [56]. Several studies have used MR analysis to assess the possible contribution of the genetically‐determined component of serum 25(OH)D and ovarian cancer [57], but this approach has yet to be applied to endometriosis. Studies have instead focused on specific SNPs within the vitamin D system, notably for the *VDR* gene and the DBP gene, also known as Group‐Specific Component (*GC*).

Three studies have assessed the possible differential representation of Fok1, Bsm1, Apa1 and Taq1 SNPs in the *VDR* gene in women with endometriosis. None of these reports described any significant association between these SNPs and the risk of endometriosis [47, 58, 59], but this may be attributed to the relatively small sample sizes (100–200 cases) in these studies and geographical localisation of sampling. One study of 154 women with endometriosis and 347 controls showed a significant association between the *VDR* Fsp1 and Fok1 SNP haplotype and endometriosis‐associated infertility, although individual *VDR* SNPs showed no association with endometriosis [60]. Similar analysis of *GC* SNPs showed no significant association between these gene variants and endometriosis [33, 58, 60]. However, two‐dimensional gel electrophoresis proteomic analysis of serum from women with different stages of endometriosis showed that the disease was associated with threefold higher abundance of DBP relative to controls [44]. Further analysis of the identified proteomic material revealed that the increased expression of DBP was due to the *GC2* variant of DBP, produced by two SNP variations [44]. The functional significance of this remains unclear, but the authors of this study speculated that the relative inability of the *GC2* form of DBP (unlike the *GC1F* form) to function as a Macrophage Activation Factor (MAF) [61], may impact the ability of this form of DBP to support innate immune surveillance, and thereby enable implantation of endometriotic tissue in the peritoneal cavity.

## 4 Key Mechanisms Linking Vitamin D and Endometriosis

Beyond its established role in the endocrinology of calcium homoeostasis and bone metabolism, vitamin D has been reported to influence a wide range of extra‐skeletal and extra‐renal tissues [25]. These effects include direct regulation of cell proliferation, apoptosis, invasion, and angiogenesis within diverse tissues, as well as indirect effects via the modulation of innate and adaptive immune responses associated with specific tissues [25]. In the setting of endometriosis, all of these different extra‐skeletal actions of vitamin D have the potential to influence the disease [62], with effects being mediated via endometrial stromal cells (ESC), endothelial cells and multiple immune cell lineages. The proposed mechanisms that support a link between vitamin D and endometriosis are primarily associated with ESC function and the immune cell infiltrates that are associated with endometriosis. The following sections of the review will explore this in more detail.

### 4.1 ESC Proliferation Invasion, and Angiogenesis

**Figure 1:**
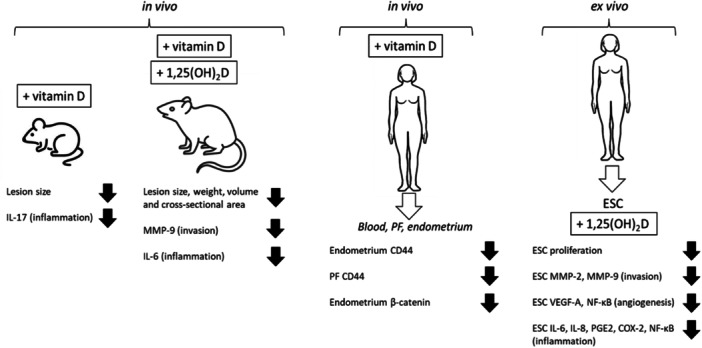
Effects of vitamin D supplementation on endometriosis. Summary of mouse, rat and human studies in vivo and ex vivo. Interleukin‐17 (IL‐17), IL‐6, IL‐8, matrix metalloproteinase‐2 (MMP‐2), MMP‐9, cluster designation 44 (CD44), endometrial stromal cell (ESC), vascular endothelial growth factor‐A (VDGF‐A), nuclear factor kappa‐B (NF‐κB), prostaglandin E2 (PGE2), cyclooxygenase‐2 (COX‐2). Peritoneal fluid (PF).

As outlined in Figure 1, studies of vitamin D and ESC have utilised in vivo supplementation of mouse and rat models of endometriosis, as well as ex vivo analysis of ESC from patients with endometriosis. In rats, studies have used both vitamin D [63, 64] and active 1,25(OH)_2_D [65] as treatment for endometriosis in these animals. These different types of vitamin D supplementation reduced the area [63], volume [65] and weight [65] of endometrial implants. These responses were associated with decreased ESC invasion in the form of lower MMP‐9 expression [65], and decreased inflammatory IL‐6 [64]. Similar suppression of endometrial implant size and expression of inflammatory IL‐17 was also observed with mice supplemented with vitamin D [66]. The overarching conclusion from these animal studies is that vitamin D has the potential to attenuate endometriosis when used in vivo.

The vast majority of studies of vitamin D and ESC function have utilised ex vivo culture of human ESC obtained from women with endometriosis. In almost all of these studies treatment was with active 1,25(OH)_2_D, and a wider array of endometriosis parameters were studied. In common with the animal in vivo studies, human ESC treated with 1,25(OH)_2_D showed decreased expression of MMP‐9 as well as MMP‐2, indicative of anti‐invasive actions [34, 67]. This anti‐invasion potential of 1,25(OH)_2_D was confirmed in another study of human ESC, which assessed the physical capacity for membrane invasion bv ESC [68]. In several of these studies 1,25(OH)_2_D was also shown to decrease ESC proliferation [34, 68], and angiogenesis in the form of suppressed vascular endothelial growth factor (VEGF) [68] or NF‐κB [34]. In endometriosis, in order for ectopic lesions to survive, they must be able to establish a new blood supply, requiring angiogenesis. In fact, the lesions are often highly vascularised [69], and VEGF has an important role in establishing and maintaining this vascularisation. VEGF is expressed in retrograde menstruation, and there is also evidence that VEGF expression is significantly higher in both the eutopic and ectopic endometrial glandular epithelium of women with endometriosis, which could contribute to the increased survival of ectopic lesions. There is also a positive correlation between vascularisation of the ectopic tissue or severity of the condition and expression of VEGF, implying that VEGF has a role in maintaining the vascularisation of ectopic tissue [70]. A wide variety of studies have assessed the impact of vitamin D on angiogenesis in different settings, notably as a factor in pregnancy and placental physiology [71], cardiovascular disease and ocular disease [72], as well as tumour development [73]. The ability of 1,25(OH)_2_D to inhibit angiogenesis in ESC by reducing expression of VEGF and associated factors such as NF‐κB suggests that the angiogenic actions of vitamin D may be crucial in its ability to suppresses implanted endometrial tissue in vivo.

To date, mechanistic studies of vitamin D and endometriosis in humans have focused primarily on ex vivo analysis of isolated ESC, and there have been only limited reports of the impact of in vivo supplementation with vitamin D in women with endometriosis. In one report, women with endometriosis were randomised to receive either standard care or standard care with 50,000 IU vitamin D per week for 12 weeks (*n* = 17 in each group) [41]. At baseline the authors showed that expression of the lymphocyte/endometriosis marker CD44 was elevated in serum, peritoneal fluid and endometrial tissue from women with endometriosis compared to healthy controls. However, this elevated expression of CD44 was then suppressed in endometrial tissue and endometrial fluid from women who received vitamin D supplementation [41]. Further analysis of tissues collected as part of this vitamin D supplementation study suggests that at least some of the effects of vitamin D on endometrial tissue is due to regulation of the β‐catenin signalling [40]. The ability of vitamin D to down‐regulate CD44 in vitro has been well described previously, notably in common cancers, where CD44 expression is elevated, and where 1,25(OH)_2_D has been reported to decrease the stem cell‐like characteristics of cancers such as ovarian tumours [74]. Interestingly, in this later study the effects of 1,25(OH)_2_D on CD44 were associated with enhanced expression of β‐catenin, suggesting that the mechanism for vitamin D regulation of CD44 in endometriosis may be different to that observed for cancers.

### 4.2 Immune Dysfunction and Inflammation in Endometriosis

In addition to dysregulation of ESC, endometriosis is also characterised by immune dysregulation and inflammation [3]. The exposure of peritoneal fluid to affected endometriosis tissue means that there is significant involvement of immune cells and their markers in the peritoneal fluid of women with endometriosis. As shown in Table 2, several cytokines and chemokines are known to be altered in endometriosis. The increase in these inflammatory factors that is observed in endometriosis does not appear to occur at the systemic level but rather within peritoneal fluid associated with the endometrial tissue. This provides a wide array of potential targets for the putative actions of vitamin D as an immunomodulator [82]. Firstly, in regulating the activities of key immune cells associated with inflammation in endometrial peritoneal fluid and, secondly, as a modulator of prostaglandins generated by the inflammatory environment that can then influence pivotal features of the endometriosis phenotype. In this regard, it is notable that a similar perspective on the immunmodulatory potential of vitamin D has been proposed for cells isolated from peritoneal fluid in the context of peritoneal dialysis for patients with chronic kidney disease [83].

**Table 2:** Cytokines elevated in the endometrial peritoneal fluid (PF) of women with endometriosis [75, 76, 77, 78, 79, 80, 81].

Cytokine with elevated levels in PF of women with endometriosis	Are there any correlations with different types of endometriosis?	Likely function(s)
IL‐1 (IL‐1α and IL‐1β)	Positive correlation with endometrial lesion size	Pro‐inflammatory: induces the synthesis of other inflammatory cytokines and COX2
TNF‐α	Positive correlation with endometrial lesion size and higher levels associated with early stages of disease	Pro‐inflammatory: induces the synthesis of other inflammatory cytokines and COX2, and is more likely to be involved in the initial establishment of lesions
IL‐6	Positive correlation with endometrial lesion size	Both pro‐inflammatory and anti‐inflammatory
IL‐10	N/A	Anti‐inflammatory: inhibits the synthesis of various inflammatory cytokines, and decreases the cytotoxicity of natural killer cells that facilitate ectopic endometrial lesion establishment
IL‐8	Higher levels associated with earlier stages of disease	Pro‐inflammatory and angiogenic chemokine. Facilitates establishment and maintenance of endometrial lesions
MCP‐1	Higher levels associated with more severe disease	Pro‐inflammatory chemokine: recruitment of immune cells
IL‐4	N/A	Anti‐inflammatory

Unbiased mass Cytometry (CyTOF) analyses have shed light on the immune cell profiles associated with endometriosis [84], showing that macrophages are the dominant immune cell type observed in endometrial peritoneal fluid. Endometriosis is associated with an elevated level of peritoneal fluid macrophages [85], which have increased activity and capacity for both cellular immunity and secreting factors. The implantation of ectopic endometrium causes peritonitis, which recruits neutrophils and macrophages to the area. Endometrial‐derived macrophages enhance the growth of ectopic lesions, whereas monocyte‐derived macrophages inhibit ectopic lesion growth and establishment [86]. Macrophages are also the pivotal immune cell type associated with the immunomodulatory actions of vitamin D. Almost all immune cell types express the VDR (resting T cells do not express VDR but induce the receptor upon immune activation), but macrophages also express the 1α‐hydroxylase enzyme that converts 25(OH)D to 1,25(OH)_2_D [87]. Thus, macrophages are the most well recognised extra‐renal source of 1,25(OH)_2_D, with this local generation of active vitamin D driving either endogenous (intracrine) responses via the macrophage VDR, or acting on adjacent immune cells that also express VDR (paracrine) [87]. This gives vitamin D substantial flexibility within any immune microenvironment, promoting antibacterial and antiviral innate immune responses within the macrophages themselves [82], in an intracrine 25(OH)D‐dependent fashion, or via further responses to macrophage 1,25(OH)_2_D by other innate immunity cells such as neutrophils [88]. In addition to this, macrophage 1,25(OH)_2_D is also able to modulate adaptive immune responses, either through intracrine regulation of antigen presentation to T cells, and subsequent modulation of T cell activation, or by paracrine actions of 1,25(OH)_2_D released by macrophages to act on VDR‐expressing activated T cells [82]. The net effect of vitamin D within the adaptive immune system is to suppress inflammatory T helper (Th) activity through modulation of Th1 and Th17 cells [89], whilst simultaneously promoting tolerogenic regulatory T cells (Treg) [90].

In addition to macrophages, other cells within the innate immune system such as dendritic cells (DC) can synthesise 1,25(OH)_2_D and also respond to the locally produced 1,25(OH)_2_D by simultaneously expressing VDR [91]. This intracrine/autocrine mode of action is finely tuned to enable functional antigen presentation by DC and associated induction of adaptive immunity T cell function, whilst allowing 1,25(OH)_2_D to promote a tolerogenic response by attenuating inflammatory responses. Specifically, there is a reciprocal relationship in DC between VDR and the 1α‐hydroxylase enzyme that generates 1,25(OH)_2_D from 25(OH)D. As DC mature to facilitate antigen presentation expression of 1α‐hydroxylase, and associated synthesis of 1,25(OH)_2_D, increases. Conversely, VDR expression decreases, suggesting that the localised synthesis of 1,25(OH)_2_D by DC is more likely to be effective on adjacent, immature DC that express higher levels of VDR [91]. In other words, this is a paracrine mode of action. The maturation of DCs appears to be central to immune dysregulation in endometriosis. Specifically, immature DCs are associated with the aberrant growth and angiogenesis associated with endometriosis, whilst mature DCs are more beneficial [92]. Unlike healthy endometrial peritoneal fluid, women with endometriosis are characterised by the presence of immature DC and decreased numbers of mature DCs [93]. This dysregulated DC response is likely to contribute to inflammation and impede the clearance of endometrial debris in the peritoneal cavity due to inefficient antigen presentation [94]. However, the aberrant DC profile associated with endometriosis will also have a profound effect on local vitamin D metabolism by decreasing the capacity for endometrial DC synthesis of 1,25(OH)_2_D. As yet, it is unclear as to whether the ability of vitamin D to induce DC tolerance is helpful or hindering in the setting of endometriosis. On one hand, it could further reduce the number of mature DCs which could facilitate the survival of ectopic lesions, worsening the dysregulation of DC outline above. On the other hand, the tolerogenic properties of 1,25(OH)_2_D could help counter the inflammation caused by DC dysregulation.

The fact that DC exhibit a similar capacity for endogenous synthesis of 1,25(OH)_2_D as macrophages, suggests that antigen presentation in general is an immune process that is highly influenced by vitamin D, with efficacy being determined by the relative levels of 25(OH)D—impaired responses in the setting of 25(OH)D‐deficiency, and enhanced responses following vitamin D supplementation and raised serum 25(OH)D. This fundamental mechanism linking vitamin D and the immune system is also likely to be central to the proposed beneficial actions of vitamin D for endometriosis. The potent effects of vitamin D on antigen presentation and subsequent T cell function reported for other inflammatory diseases are associated with altered expression of a wide array of cytokines [95], and these anti‐inflammatory actions of vitamin D are likely to be pivotal in its proposed beneficial actions on endometriosis. In support of this, studies in vivo using a mouse model of endometriosis (induced following syngeneic injection of endometrial fragments into female mice) have shown that the synthetic 1,25(OH)_2_D analog elocalcitol suppresses macrophage recruitment and inflammatory cytokine levels [96]. This anti‐inflammatory action of the vitamin D analog was associated with decreased progression of endometrial disease as determined by endometrial lesion weight [96].

Endometriosis is also characterised by distinct changes in cells from the adaptive immune system. In the peritoneal fluid around ectopic endometrial lesions, there is an overall increase in T helper‐cells, which secrete elevated levels of cytokines. The phenotypes of endometriosis T‐helper cells favours T2 helper cells (Th2) and T17 helper cells (Th17) rather than T1 helper cells (Th1) [94, 97]. In other tissue immune settings, 1,25(OH)_2_D has been shown to promote downregulation of both Th1 and Th17 cells [82] and would therefore promote potential beneficial effects in the setting of endometriosis. However, it should also be recognised that studies of other inflammatory diseases such as rheumatoid arthritis have shown that establishment of the initial inflammation associated with the disease leads to an inherent insensitivity to 1,25(OH)_2_D [98]. This is due, in part, to the increased proportion of memory T cells in disease affected tissue relative to the periphery and suggests that 1,25(OH)_2_D may be more advantageous in preventing diseases such as endometriosis, rather than as a treatment once the disease is established.

**Figure 2:**
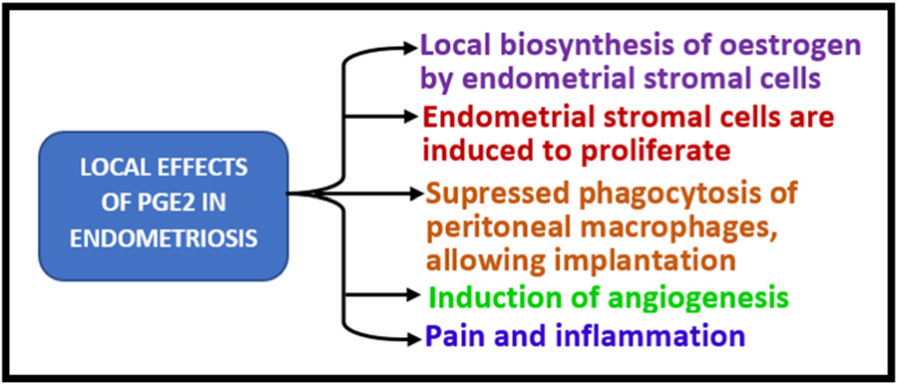
Local effects of PGE2 in endometriosis.

In addition to its actions on antigen presentation, T cell function and inflammatory cytokine expression, vitamin D can also influence inflammatory mechanisms outside the central immune system. Inflammatory cytokines such as IL‐1 and tumour necrosis factor alpha (TNF‐⍺) also induce cyclooxygenase‐2 (COX2) expression, which upregulates prostaglandin 2 (PGE2) synthesis primarily by endometrial glandular epithelium, but also by associated inflammatory macrophages. Prostaglandins have been implicated in normal endometrial function and proposed as markers of implantation [99]. However, endometriotic tissue is characterised by much higher levels of COX2 and PGE2 than unaffected endometrial tissue [100, 101], suggesting that PGE2 contributes to the inflammatory pathophysiology of endometriosis (Figure 2). It is likely that the release of these pro‐inflammatory factors in endometriosis is local and not peripheral, because only endometriosis peritoneal macrophages were found to have upregulated COX2, not peripheral macrophages [102].

In preclinical models using prostate cancer cells, 1,25(OH)_2_D has been shown to decrease expression of COX2, whilst stimulating catabolism of prostaglandins via upregulation of 15‐hydroxyprostaglandin dehydrogenase (15‐PGDH), thereby supressing PGE2 levels [103]. Similarly, studies of breast tissue from healthy women have shown that vitamin D supplementation in vivo results in decreased expression of mRNA for COX2 and PGE2 levels [104]. Thus, it seems likely that vitamin D may act as a key regulator of COX2 and PGE2 in endometrial tissue and endometriosis. In addition, the observation that vitamin D supplementation, leading to increased circulating levels of 25(OH)D is sufficient to promote COX2/PGE2 suppression, suggests that localise, tissue‐specific conversion of 25(OH)D to 1,25(OH)_2_D is central to the anti‐inflammatory actions of vitamin D. With this in mind, it is interesting to note that in macrophages from other tissue sites such as synovial fluid, PGE1 and PGE2 have been shown to potently suppress the ability of these cells to convert inactive 25(OH)D to active 1,25(OH)_2_D, suggesting that the onset of endometriosis compromises the ability of vitamin D to modulate normal macrophage function [105]. This may explain, in part, other observations of macrophage function in endometriosis. Endometriosis is associated with an elevated levels of peritoneal macrophages [85], which have increased activity and capacity for both cellular immunity and cytokine and prostaglandin synthesis. However, disease‐associated endometrial‐derived macrophages enhance the growth of ectopic lesions, whereas non‐involved peripheral blood monocyte‐derived macrophages inhibit ectopic lesion growth and establishment [86].

## 5 Conclusions and Future Studies

Despite the prevalence of endometriosis as a gynaecological disorder, its aetiology remains unclear and diagnostic and treatment options continue to be poor. In recent years, in common with many other chronic diseases, there has been growing interest in a possible role for vitamin D as both a marker and therapeutic option for endometriosis. The gold standard for diagnosis, laparoscopy, is an invasive procedure, and there are not any minimally invasive techniques which can confirm a diagnosis yet, such as a serum biomarker [106]. Treatment options for endometriosis are mainly to alleviate symptoms and delay recurrence post‐surgery by suppressing ovarian function, rather than being curative [107]. Thus, effective and convenient alternative approaches to the management of endometriosis are clearly required, with vitamin D supplementation being a potential candidate strategy. Current studies suggest that serum 25(OH)D deficiency is linked to endometriosis severity, and thus measurement of 25(OH)D as an endometriosis marker could be a useful adjunct to the current analyses used to assess women with endometriosis. This at least provides the option of vitamin D supplementation for those women observed to be vitamin D deficient and would be justified based on conventional requirements for musculoskeletal health. However, the limited number of vitamin D supplementation studies carried out to date suggest that there may also be other benefits for women with endometriosis, notably with respect to inflammation and pain reduction. Further studies are required to determine the precise levels of serum 25(OH)D associated with improvement in endometriosis symptoms (and the vitamin D supplementation dose required to achieve this). More challenging studies could also be carried out to assess the possible benefits of improved vitamin D status in protecting against the onset of disorders such as endometriosis. As outlined earlier in this review, to successfully execute these types of trials it will be important to better define the immunomodulatory function of vitamin D in endometriosis, in a similar fashion to the many studies already reported for vitamin D and infectious and autoimmune disease. However, in view of the relative safety and cost of vitamin D supplementation, there is potentially much to be gained by continued exploration of the wider benefits of vitamin D for endometriosis and women's reproductive health in general.

## Conflicts of Interest

The authors declare no conflicts of interest.

## Data Availability

Data sharing is not applicable to this article as no datasets were generated or analysed during the current study.
